# ASCL1 Is Involved in the Pathogenesis of Schizophrenia by Regulation of Genes Related to Cell Proliferation, Neuronal Signature Formation, and Neuroplasticity

**DOI:** 10.3390/ijms242115746

**Published:** 2023-10-30

**Authors:** Dmitrii A. Abashkin, Dmitry S. Karpov, Artemii O. Kurishev, Ekaterina V. Marilovtseva, Vera E. Golimbet

**Affiliations:** 1 Mental Health Research Center, Kashirskoe Sh., 34, Moscow 115522, Russia; 2 Center for Precision Genome Editing and Genetic Technologies for Biomedicine, Engelhardt Institute of Molecular Biology, Russian Academy of Sciences, Vavilov Str. 32, Moscow 119991, Russia

**Keywords:** ASCL1, schizophrenia, bipolar disorder, SH-SY5Y, transcriptome, CRISPR/Cas9

## Abstract

Schizophrenia (SZ) is a common psychiatric neurodevelopmental disorder with a complex genetic architecture. Genome-wide association studies indicate the involvement of several transcription factors, including ASCL1, in the pathogenesis of SZ. We aimed to identify ASCL1-dependent cellular and molecular mechanisms associated with SZ. We used Capture-C, CRISPR/Cas9 systems and RNA-seq analysis to confirm the involvement of ASCL1 in SZ-associated pathogenesis, establish a mutant SH-SY5Y line with a functional *ASCL1* knockout (ASCL1-del) and elucidate differentially expressed genes that may underlie ASCL1-dependent pathogenic mechanisms. Capture-C confirmed the spatial interaction of the *ASCL1* promoter with SZ-associated loci. Transcriptome analysis showed that *ASCL1* regulation may be through a negative feedback mechanism. ASCL1 dysfunction affects the expression of genes associated with the pathogenesis of SZ, as well as bipolar and depressive disorders. Genes differentially expressed in ASCL1-del are involved in cell mitosis, neuronal projection, neuropeptide signaling, and the formation of intercellular contacts, including the synapse. After retinoic acid (RA)-induced differentiation, ASCL1 activity is restricted to a small subset of genes involved in neuroplasticity. These data suggest that ASCL1 dysfunction promotes SZ development predominantly before the onset of neuronal differentiation by slowing cell proliferation and impeding the formation of neuronal signatures.

## 1. Introduction

Schizophrenia (SZ) is a common psychiatric disorder characterized by clinical behavioral symptoms and underlying brain dysfunction [1]. The genetic component is a major contributor to the development of SZ, and its heritability is generally estimated to be between 30% and 80% [2]. The complex genetic architecture of SZ includes common alleles, rare gene mutations, copy number variations, deletions, and chromosomal translocations. Genome-wide association studies (GWAS) have identified genetic variants associated with SZ [3] that have served as a comprehensive resource for further mechanistic studies [4]. GWAS [3,5,6] and recent genetic studies [7] linked a number of transcription factors (e.g., TCF4, PRDM14, POU5F1, TEAD1, ZEB2, FOXP1, ZNF804A, etc.), including ASCL1, with SZ. However, to date, there is no evidence for a functional connection between ASCL1 dysfunction and SZ pathogenesis.

ASCL1 (previously called *MASH1*, mammalian achaete–scute homologue 1) is a class II basic helix–loop–helix (bHLH) neuron-specific transcription factor (TF), known to be involved in the development of the human central nervous system [8,9]. Jane Johnson et al. cloned *ASCL1* cDNA from embryonic rat nervous systems due to its amino acid sequence similarity to the bHLH proteins of *Drosophila melanogaster* achaete–scute complex and revealed that *ASCL1* (*MASH-1*) had a temporal expression in the mid-gestational period during days 10.5–11.5, which declined at day 20.5 and became undetectable in the adult brain. Interestingly, *ASCL1* overexpression in non-neural cell lines (NIH3T3, PC12, and HeLa) caused no obvious phenotype [10]. *ASCL1* expression is spatially restricted to neural precursors in the forebrain, where its level declines along with the neurons’ maturation [11]. This observation agrees with the role of achaete–scute complex in *Drosophila melanogaster*, where it primes the selection of neuroblasts among the unspecified ectodermal cells in the process called lateral inhibition [12].

ASCL1 forms a functional heterodimer with other bHLH E-proteins in order to bind to the so-called E-box (5′-CANNTG-3′). One of its binding partners is a class A TF neurogenin-2 (NEUROG2, an orthologue of *D. melanogaster* gene *atonal*), which induces the expression of regulators of transcription, signal transduction, and cytoskeletal rearrangement for neuronal differentiation and migration. Overexpression of either ASCL1 or NEUROG2 in astrocytes leads to their transdifferentiation into functional neurons [13,14]. Although these two TFs tend to bind different sets of promoters, they can substitute each other in some regions of the nervous system [15]. Due to its ability to interact with ATP-dependent BAF SWI/SNF chromatin remodeling complex, ASCL1 also plays a role in the regulation of chromatin structure, primarily at its target promoters, in embryonic stem cells and neural progenitors, as well as in glioma cell lines [16]. Finally, ASCL1 was shown to interact with both WNT and NOTCH signaling pathways to control neuronal differentiation and the tumorigenicity of glioma cell lines, genetically engineered glioma mouse models, and brain tumors of patient-derived xenograft (PDX)-GBM [17]. Thus, current evidence suggests that ASCL1 is an inducer of neuronal differentiation with a temporally restricted period of activity and mediates extensive chromatin remodeling at target genes.

Here, we showed that the *ASCL1* promoter spatially interacts with SZ-associated loci located in C12orf42 using Capture-C. To further elucidate the possible ASCL1-mediated cellular and molecular mechanisms associated with SZ, we generated an SH-SY5Y cell line with a functional *ASCL1* knockout using the CRISPR/Cas9 system. We then compared the transcriptomes of the original cell line, the cell line expressing non-targeting sgRNA (NTC), and the mutant cell line before and after differentiation into neuron-like cells.

## 2. Results

### 2.1. Interaction of the ASCL1 Promoter with SZ-Associated Loci

**Figure 1:**
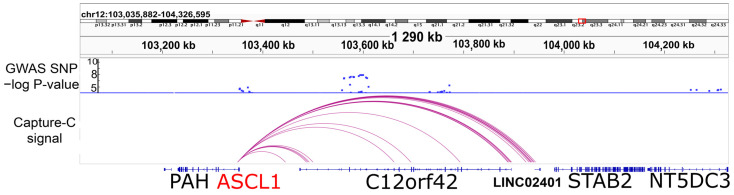
Capture-C revealed the interaction of the *ASCL1* promoter with the SZ-associated locus C12orf42. The −log(*p*-value) data of SZ-associated SNPs found in the PGC-3 GWAS [3] (**upper panel**) and Capture-C data for SH-SY5Y (CHiCAGO output, **lower panel**) are presented. Red box indicates the position of a gene locus on a chromosome.

Previously, we developed a modified Capture-C protocol for high-resolution analysis of promoter interactomes [18]. To search for possible spatial interactions of the *ASCL1* promoter with SZ-associated loci, we used the neuroblastoma cell line SH-SY5Y. This cell line exhibits the phenotype of immature neurons, expressing the dopamine, gamma-aminobutyric acid, glutamate, and acetylcholine receptors DRD2, GABRA2, GRIA4, and CHRNA3. The morphology of SH-SY5Y also resembles neurons, as it contains branching neurites [19]. Using the Capture-C method in SH-SY5Y, we detected the spatial chromatin interactions of the *ASCL1* promoter with the non-coding regions of C12orf42 introns carrying SZ-associated loci (Figure 1). According to the Psychiatric Genomics Consortium (PGC-3) GWAS SZ, SNPs with *p*-values below 10^−5^ are present in the CDS of *ASCL1*, and those with *p*-value below 10^−8^ are present in the intronic regions of C12orf42 (raw data are available at https://pgc.unc.edu/for-researchers/download-results, accessed on 20 October 2023, Table S1) [3]. Moreover, in previous GWAS studies, C12orf42 appeared among 108 genes associated with SZ [20].

Given the role of ASCL1 in early neurodevelopment, these data suggest that *ASCL1* is a promising candidate gene whose dysfunction leads to early events predisposing to SZ or other neuropsychiatric disorders.

### 2.2. Generation of the Mutant SH-SY5Y Line with ASCL1 Partial Deletion 

**Figure 2:**
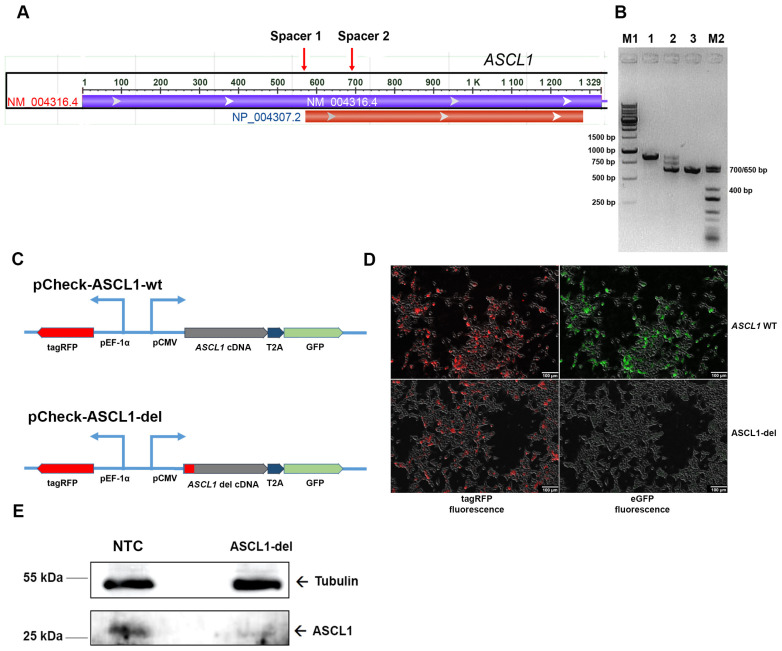
Generation of the SH-SY5Y cell line with impaired synthesis of ASCL1 protein. (**A**) Position of sgRNAs to introduce a small deletion in the 5’-region of the *ASCL1* gene. The first exon containing the *ASCL1* ORF is shown. (**B**) PCR analysis of NTC cells with intact *ASCL1* (lane 1), a mixed cell population prior to cell cloning (lane 2), and a clone with a partial deletion in the *ASCL1* gene (lane 3). M1, 1 kb DNA ladder (Sibenzyme, Russia); M2, TriDye Ultra Low Range DNA ladder (NEB, USA). (**C**) Schemes of reporter constructs to assess ASCL1 production from wild-type and mutant cDNAs. (**D**) Fluorescence microscopy of HEK293T cells transfected with pCheck-ASCL1-wt (top panel) and pCheck-ASCL1-del (bottom panel) plasmids. (**E**) Detection of ASCL1 protein in NTC and ASCL1-del cell lines by Western blotting. ASCL1 was stained with rabbit polyclonal antibodies. α-tubulin was used as a reference.

To elucidate the role of *ASCL1* dysfunction in SZ-related mechanisms, we obtained an SH-SY5Y line with a mutant *ASCL1* gene. Using the CRISPR/Cas9 system, we introduced a partial deletion affecting the 5’-untranslated region of *ASCL1* and the start codon (Figure 2A). The genomic coordinates of the expected deletion are chr12:102,958,236–102,958,352 according to the human genome version hg38. We expected the mutant gene to be transcribed into truncated transcripts that would not be translated, resulting in a functional knockout of *ASCL1*. To account for possible changes in the cell transcriptome caused by the transduction of lentiviral vectors [21], we also obtained a control cell line stably expressing sgRNA not directed against any of the targets in the human genome (non-target control, NTC).

To introduce the *ASCL1* deletion, SH-SY5Y cells were sequentially transduced with lentiviral vectors, with one containing the SpCas9 gene under tight control of the tetracycline-inducible promoter (eGFP-Puro chimeric marker) and the others carrying guide RNAs (tagRFP marker). After sorting cells expressing both eGFP and tagRFP, the presence of a deletion in *ASCL1* in the mixed population was verified by PCR (Figure 2B). Wild-type *ASCL1* yielded a 860-base-pair (bp)-long PCR product (Figure 2B, lane 1), whereas mutant alleles with the same primers yielded several short PCR products (Figure 2B, lane 2). Subsequent selection using limiting dilutions yielded a pure clone with a 220 bp homozygous deletion giving a 640 bp PCR product (Figure 2B, lane 3).

To verify the absence of ASCL1 synthesis in the mutant allele, we cloned the open reading frames from the wild-type and mutant cDNAs into the pCheck reporter vector, which was designed to constitutively express tagRFP from the strong EF-1α promoter and to assess translation of the gene of interest (GOI) cloned under the control of the pCMV promoter in a frame with T2A-eGFP lacking the first methionine. Thus, if the GOI is translationally competent, GFP should be expressed, and its signal should be detected; otherwise, no GFP signal can be detected.

ORFs from cDNA of wild-type and mutant ASCL1 were cloned into pCheck to produce the plasmids pCheck-ASCL1-wt and pCheck-ASCL1-del (Figure 2C). The plasmids were transfected into the HEK293T cell line, and the fluorescence of the reporter proteins was visualized in an unsorted cell population. As a result (Figure 2D), the wild-type *ASCL1* allele produced a strong GFP signal, while the mutated *ASCL1* allele did not produce a significant GFP signal. These results strongly suggest that translation of the mutant ASCL1 mRNA is impaired.

To further confirm the impaired synthesis of ASCL1 protein in the mutant SH-SY5Y clone, we used Western blot analysis. According to the results (Figure 2E), the level of ASCL1 is significantly reduced and the size of the detectable band appears to be slightly lower compared with the wild-type protein in the NTC control. These results correlate well with those obtained in the reporter system (Figure 2D) and indicate that ASCL1 synthesis is disrupted in the mutant line.

### 2.3. General Characterization of Transcriptomes of SH-SY5Y Cell Lines

Because ASCL1 regulates neurodevelopmental processes by acting as a TF, we expect that disruption of ASCL1 function would result in the altered expression of genes that are, in turn, associated with SZ pathogenesis. We therefore compared the transcriptomes of ASCL1-del and NTC cell lines cultured under standard conditions or differentiated into neurons after retinoic acid (RA) treatment using RNA-seq of RNA fractions enriched with polyadenylated transcripts.

**Figure 3:**
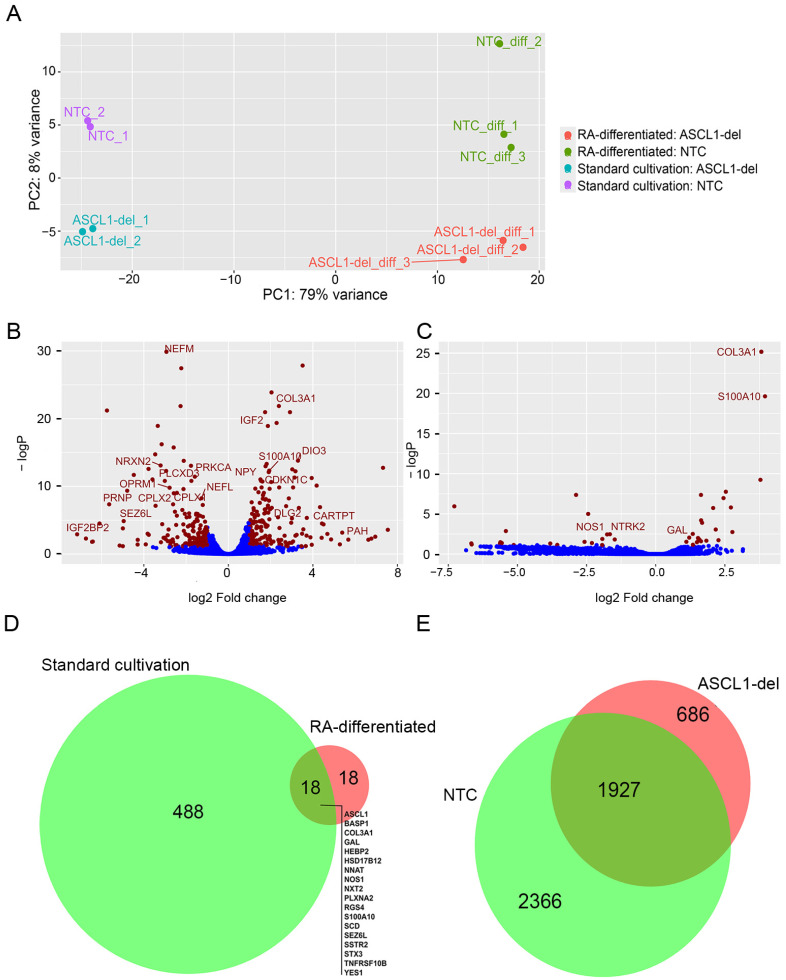
(**A**) PCA plot of normalized counts using stabilizing variant transformation. Volcano plots of DEGs identified by RNA sequencing analysis comparing derivatives of the SH-SY5Y cell line (ASCL1-del vs. NTC) cultured under standard conditions (**B**) or (**C**) after RA-induced differentiation. Blue dots indicate genes whose expression level changes are not statistically significant or are below the fold change threshold. (**D**) Venn diagram [22] for the data presented in (**B**,**C**). The list of genes represents DEGs common to standard and RA-treated conditions. (**E**) Venn diagram for DEGs in NTC (standard cultured) vs. NTC (RA-differentiated) and ASCL1-del (standard cultured) vs. ASCL1-del (RA-differentiated) ASCL1-del.

A principal component (PCA) analysis using the lists of differentially expressed genes (DEGs) identified by DESeq2 (Tables S2 and S3) showed that the replicates segregated well into four groups (Figure 3A). PCA also revealed that most of the variance (~79%) was associated with RA-induced differentiation and only 8% with *ASCL1* mutation. It should be noted that the distance between biological replicates of the NTC and mutant cell line increases after cell differentiation, indicating an increase in variance in gene expression.

We found 506 genes that were significantly (adjusted *p*-value < 0.05) differentially expressed in ASCL1-del compared to NTC SH-SY5Y in the native state (Figure 3B, Table S2) and only 36 DEGs after RA-induced differentiation (Figure 3C, Table S3). In other words, ASCL1 dysfunction causes the most significant changes in the transcriptome of SH-SY5Y cells in the native state. In the process of RA-induced cell differentiation, there is a significant compensation of ASCL1-dependent transcriptome changes. Half of the DEGs detected in differentiated cells (18 genes) coincide with the DEGs detected in native cells (Figure 3D). During RA-induced differentiation of SH-SY5Y into neurons, 4293 and 2613 genes had altered expression levels in NTC and ASCL1-del, respectively (Figure 3E, Tables S4 and S5). The DEG ratio (4293/2613) was not as significant after RA treatment as under normal conditions (506/36, Figure 3D), and this further confirms that ASCL1 has little effect on the cell differentiation process.

Because we made a partial deletion that preserves transcription of the *ASCL1* gene, we found in the RNA-seq data that there is a significant increase in *ASCL1* expression in the ASCL1-del mutant line. The results of RT-qPCR (Figure S1a) confirm the RNA-seq data and indicate that ASCL1 can serve as a direct or indirect repressor of its own gene, which means that *ASCL1* expression is regulated by a negative feedback mechanism.

We found a subset of genes such as *NOS1*, *NRAP*, *GUCY1A2*, *GUCY1A2*, *CLSTN2* and *NTRK2* that are silenced in the undifferentiated SH-SY5Y cell line but activated upon RA treatment (Table S3). However, their expression level in the ASCL1-del line is lower than in the NTC control, despite the fact that *ASCL1* itself is repressed upon RA treatment (Table S3). These data suggest the existence of neuron-specific genes that can be activated indirectly through ASCL1-dependent activation of other TFs.

### 2.4. Differential Changes in the Transcriptome in Undifferentiated SH-SY5Y Cell Lines 

We next performed a separate comparative analysis of the transcriptomes of the undifferentiated ASCL1-del mutant line and the control NTC.

#### 2.4.1. Analysis of Transcription Factor Enrichment

Our data (Section 2.3) suggest that other TFs are also involved in the regulation of ASCL1-dependent genes. Moreover, among DEGs derived to control NTC and ASCL1 deletion, approximately half (249 genes) were significantly upregulated and the other half (257 genes) were significantly downregulated. These data suggest that the role of ASCL1 in transcriptional regulation may depend on direct or indirect interactions with other TFs.

**Figure 4:**
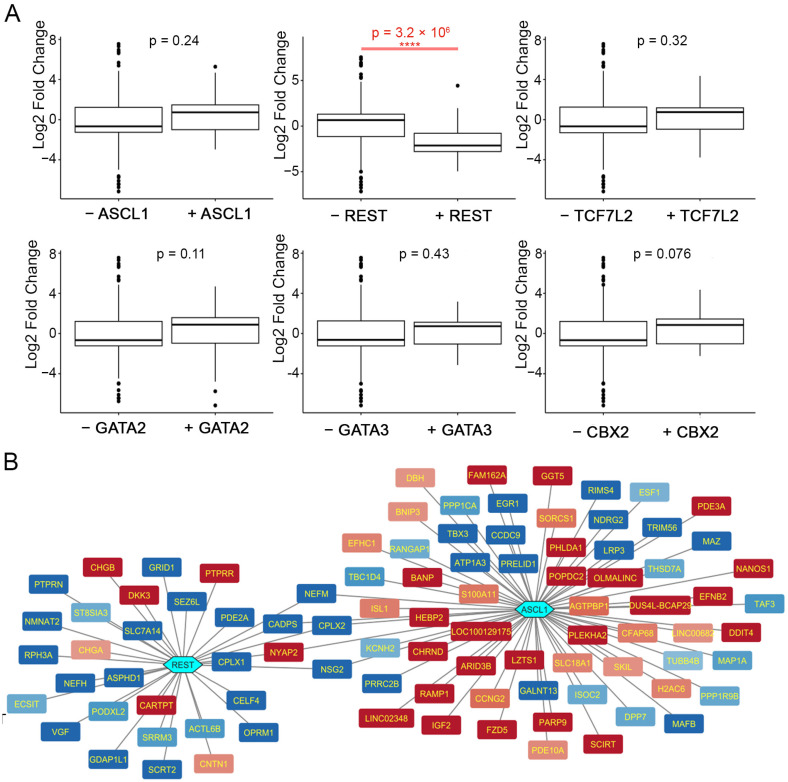
(**A**) Distributions of DEG log2 fold change, depending on the presence/absence of TF binding. (**B**) Putative REST- and ASCL1-targets regulated directly in undifferentiated SH-SY5Y cell lines.

To search for possible TFs interacting with ASCL1, the first step was to analyze DEGs with TF enrichment using available ChIP-seq ENCODE data. The TFs REST (RE1-silencing transcription factor), TCF7l2 (transcription factor 7 like 2, also known as TCF4), GATA3 (GATA-binding protein 3), GATA2 (GATA-binding protein 2), and CBX2 (chromobox 2) were significantly enriched (FDR < 0.05) among the identified DEGs (Table 1). In the second step, the association between ChIP-seq data of ASCL1 and other TFs (Table S6) with the expression level of DEGs was analyzed (Figure 4A). A nonparametric Wilcoxon test was applied to determine the significance of the effect of TF binding on the log2 fold change among DEGs. It appeared that ASCL1 binding did not correlate with the expression level of DEGs, supporting the assumption that ASCL1 affects the expression of most of its target genes indirectly. Only REST binding significantly correlated with the log2 fold change of DEGs (Figure 4A).

Since REST binding correlates with decreased expression levels of DEGs (*p*-value = 5.371 × 10^−6^), these data suggest that REST acts as an antagonist of ASCL1 in undifferentiated SH-SY5Y cells. At the same time, only 5 matches were found among 31 proposed direct targets of REST and 67 targets of ASCL1 (Figure 4B), indicating a predominantly indirect interaction between REST and ASCL1.

#### 2.4.2. Enrichment Analyses by Disease and Gene Ontology

**Figure 5:**
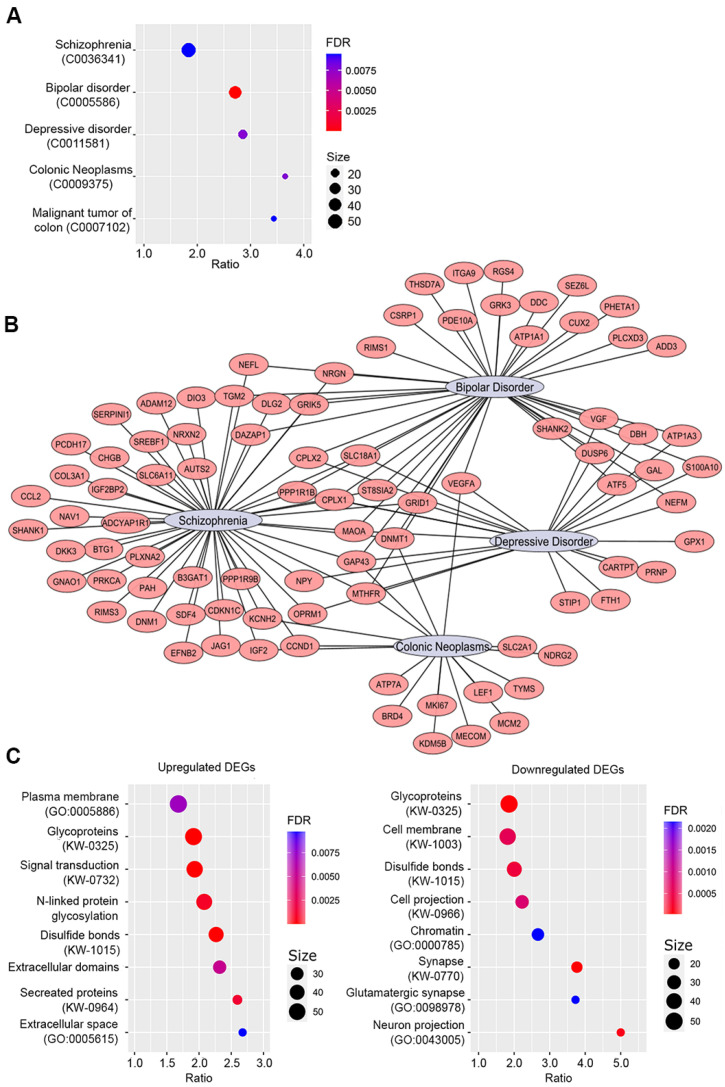
ASCL1 dysfunction in undifferentiated cells is linked to SZ pathogenesis through effects on neuronal phenotype, synapse formation, and function. (**A**) Enrichment for the disease-associated genes among DEGs based on the DisGeNET database. (**B**) Gene-disease association network. (**C**) Gene Ontology (GO) and UniProtKB (KW) categories, enriched in upregulated and downregulated DEG lists.

To test whether ASCL1 dysfunction in undifferentiated cells is connected to SZ pathogenesis, we performed an enrichment analysis of gene–disease associations using the DisGeNET [23] database and the DAVID online enrichment service [24]. The total SH-SY5Y transcriptome we obtained was used as a background dataset. Our results (Figure 5A, Table S7) indicate that DEGs are significantly (FDR < 0.01) associated with psychiatric disorders such as SZ, bipolar disorder (BP), and depressive disorder. The highest number of genes (50) was associated with SZ. A more detailed analysis (Figure 5B) showed that some DEGs are common to several diseases. For example, *CPLX1*, *CPLX2*, *GAP43*, *GRID1*, *NEFL*, *NRGN*, *PPP1R1B*, *SLC18A1*, and *ST8SIA2* are common to SZ and BP. This is consistent with the fact that SZ and BP are genetically related [25]. Thus, our results suggest that our cellular model is suitable for a deeper analysis of the ASCL1-dependent mechanisms associated with SZ.

In addition to neuropsychiatric disorders, a number of DEGs are associated with malignant neoplasms (Figure 5A). These data are in good agreement with earlier findings on the involvement of ASCL1 in neuroblastoma development through the regulation of key TF and cell cycle genes [26].

To identify biological processes, structures, and pathways dysregulated in ASCL1-deprived cells, a GO enrichment analysis of up- and down-regulated genes was performed using the DAVID enrichment online server. The most interesting affected categories are “cell projection”, “neuronal projection”, “plasma membrane”, “cell membrane”, “signal transduction”, and “glutamatergic synapse”, indicating that the ASCL1 mutation affects neuronal phenotype formation and synapse function.

Overall, the transcriptomic data support the validity of using SH-SY5Y as a model to study the ASCL1-dependent mechanisms associated with SZ pathogenesis and suggest that disruption of ASCL1 function affects the cytoplasmic membrane proteome, resulting in a partial loss of neuronal signatures and synaptic dysfunction.

#### 2.4.3. Gene-Set Enrichment Analysis (GSEA) of DEGs

To obtain information about the sets of biologically relevant genes affected by ASCL1 dysfunction, we applied the GSEA assay to the list of DEGs. Using gene sets provided in the Molecular Signature Database [27], we expected GSEA to identify the most similar set(s) of biologically relevant genes that can provide insight into which cellular processes are most matched to our DEG profile.

**Figure 6:**
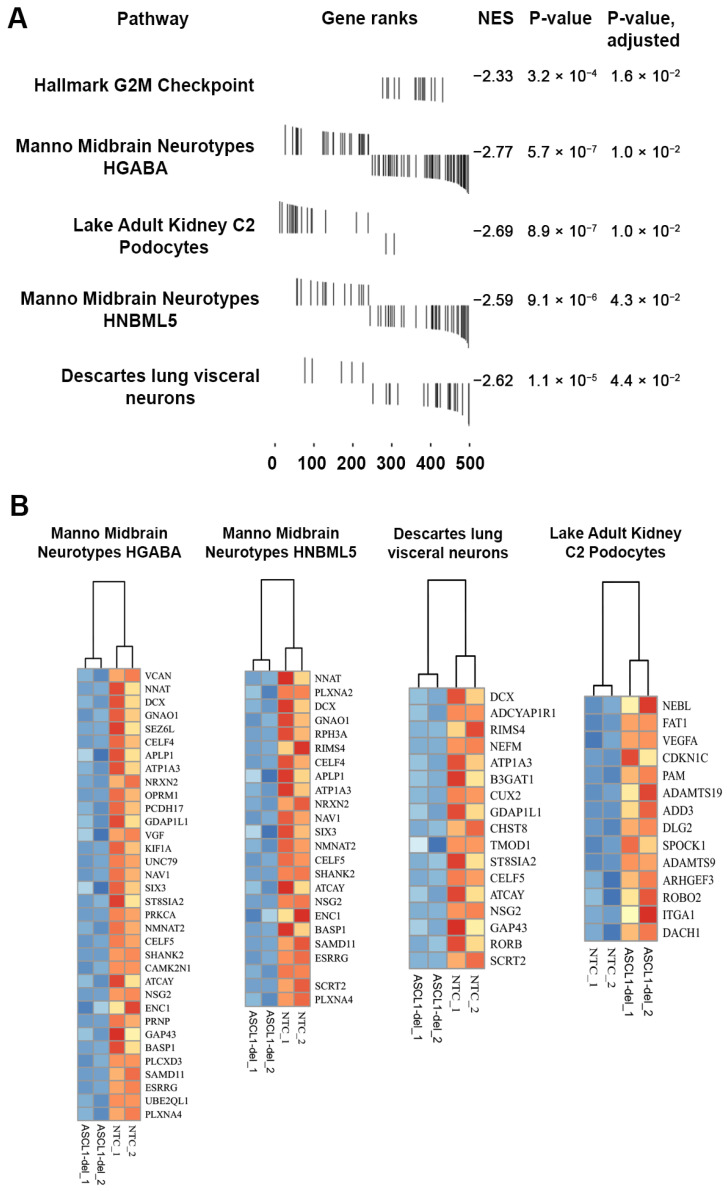
(**A**) GSEA results obtained for the DEG list (ASCL1-del genes versus NTC). NES is the enrichment score. (**B**) Heatmaps of centered normalized gene expression of ASCL1-del versus NTC for leading-edge genes from GSEA results created using the ‘pheatmap’ package R.

GSEA returned four statistically significant hits (adjusted *p*-value < 0.05) from the Molecular Signature Database (version v2023.1.Hs) and one hit from the Hallmark gene set [28] (Figure 6A). The Hallmark gene set hit was a G2M checkpoint (HALLMARK_G2M_CHECKPOINT), which contained 15 genes in the leading edge: *TACC3, MCM2, MCM5, TNPO2, UBE2S, CCND1, LMNB1, SMC4, NCL, DDX39A, NASP, MKI67, INCENP, PLK1*, and *ATF5*. These genes are downregulated in ASCL1-del cells. The biological relevance of this finding is supported by the reduced growth rate of ASCL1-del cells that we observed in normal culturing and reported previously [29], as well as earlier results showing that ASCL1 is a regulator of cell cycle genes [17].

Hits from the Molecular Signature Database are associated with characteristic gene sets of different cell types (Figure 6B). Three of these relate to neuronal gene sets: Descartes fetal visceral lung neurons, Manno midbrain HNBML5 neurotypes, and Manno midbrain HGABA neurotypes. The combined gene list for these three neuronal gene sets contains 45 down-regulated genes: *NSG2*, *ESRRG*, *UBE2QL1*, *KIF1A*, *PRKCA*, *NRXN2*, *BASP1*, *PLCXD3*, *DCX*, *CELF4*, *OPRM1*, *PRNP*, *NNAT*, *CAMK2N1*, *PLXNA4*, *VGF*, *ATP1A3*, *UNC79*, *SEZ6L*, *PCDH17*, *SIX3*, *GDAP1L1*, *ST8SIA2*, *SHANK2*, *ENC1*, *GAP43*, *ATCAY*, *CELF5*, *NAV1*, *VCAN*, *SAMD11*, *GNAO1*, *APLP1*, *NMNAT2*, *RPH3A*, *PLXNA2*, *SCRT2*, *RIMS4*, *NEFM*, *CHST8*, *ADCYAP1R1*, *RORB*, *CUX2*, *TMOD1*, and *B3GAT1*. This list shows highly significant overlap with disease-associated DEGs (*p*-value = 0.002, representation factor 2.5). Interestingly, REST targets were also overrepresented in this set (*CELF4*, *RPH3A*, *SCRT2*, *GDAP1L1*, *NEFM*, *OPRM1*, *NMNAT2*, *SEZ6L*, *NSG2*, and *VGF*; *p*-value 1.25 × 10^−4^; representation factor 3.6). Based on ChIP-seq data, *ATP1A3*, *NEFM*, *NSG2*, and *RIMS4* appeared to be possible direct targets of ASCL1. Although these three gene sets confirmed the molecular features of neuronal cells in ASCL1-del, we did not observe any noticeable changes in cell morphology under light microscopy compared to NTC.

The fourth set of genes (14 leading edge matches: *NEBL*, *FAT1*, *VEGFA*, *CDKN1*, *PAM*, *ADAMTS19*, *ADD3*, *DLG2*, *SPOCK1*, *ADAMTS9*, *ARHGEF3*, *ROBO2*, *ITGA1*, and *DACH1*) corresponding to podocytes (C2 podocytes of the adult kidney) was significantly upregulated in the ASCL1-del mutant line compared to NTC. Nine proteins encoded by these genes are localized at the plasma membrane or in the extracellular space. SPOCK1 is considered a mediator of the epithelial–mesenchymal transition in various cell lines [30,31]. It can work in concordance with upregulated *VEGFA* [32]. *VEGFA*, encoding the vascularization growth factor VEGF, is highly expressed in various neuroblastoma cell lines [33]. *ITGA1*, *FAT1*, and *ROBO2* act as counterparts in intercellular adhesion and cell migration. 

GSEA revealed pleiotropic activity of ASCL1 in SH-SY5Y cells. First, ASCL1 can regulate the G2M transition of the cell cycle. Second, ASCL1 is involved in neuronal signature formation, the disruption of which is part of the pathogenesis of psychiatric diseases. Third, ASCL1 can reshape the plasma membrane proteome and control the formation of intercellular contacts.

#### 2.4.4. Gene Interaction Network Analysis 

**Figure 7:**
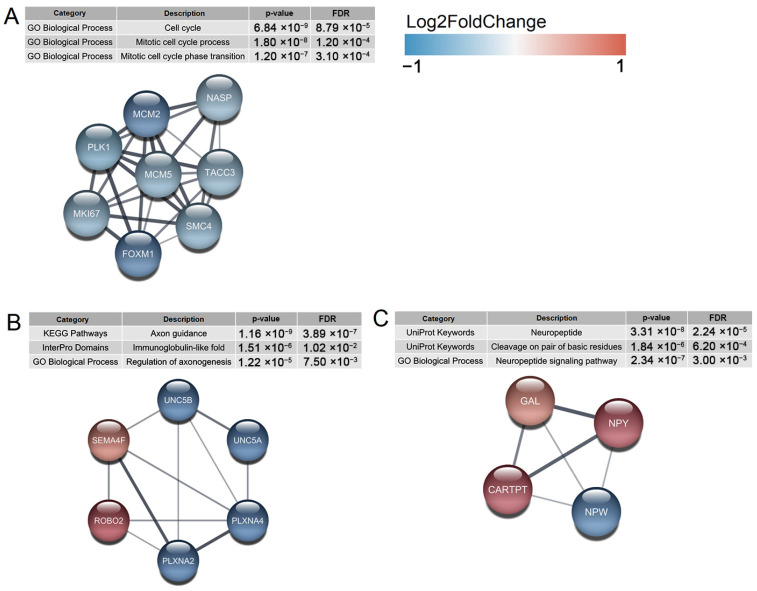
STRING database interactions of the proteins constructed from DEG list (ASCL1-del genes versus NTC). (**A**) Protein complex involved in cell cycle regulation. (**B**) Protein complex involved in axonogenesis. (**C**) Protein complex involved in neuropeptide Y signaling.

We used the STRING database (StringApp, Cytoscape) [34] to obtain more in-depth information on protein complexes and other types of interactions of proteins encoded by DEGs. The genes of the G2M cluster formed a clique subgraph with high co-expression values in STRING scores (Figure 7A, Table S8). Downregulation of this cluster upon ASCL1 dysfunction strongly suggests cell cycle delay in the mutant cell line. Another detected cluster of interacting proteins is related to axonogenesis and axon guidance (Figure 7B). Deregulation of the corresponding genes suggests that these processes may be negatively disrupted in neurons as well. As previously found, deletion of *ASCL1* leads to increased levels of gene expressing neuropeptide Y(NPY) [29]. Our results also support this finding (Figure 7C). NPY imbalance has been implicated in the development of metabolic diseases, including obesity, glucose tolerance, hypertension and atherosclerosis [35], but the functional significance and association with SZ remain unclear.

### 2.5. Changes in the Transcriptome of SH-SY5Y Lines during RA-Induced Differentiation Procedure

Since ASCL1 is involved in neurodevelopment, it is of great interest to evaluate its ability to influence the process of neuronal differentiation. For this purpose, we treated both NTC and ASCL1-del cell lines with RA to induce differentiation into neuron-like cells. The success of the differentiation process was confirmed by light microscopy, which clearly shows neurite formation in both cultures (Figure S2). The transcriptomes of differentiated cells were then sequenced and compared. As previously noted, 4293 and 2613 DEGs associated with cell differentiation were identified in NTC and ASCL1-del (Figure 3E).

To confirm the cellular response to RA treatment, we searched for RA-responsive TFs using CheA3 analysis against the ENCODE database for the DEGs NTC_RA. We found 73 TFs with FDR < 0.05, and we found RXRA (FDR = 0.0185, odds ratio = 1.763) encoding retinoid X receptor Alpha in the top ten by odds ratio. Our dataset contains 33 DEGs that may be targets of RXRA (Table S9). To identify DEGs directly regulated by RXRA, we searched for common retinoic acid response element (RARE) motifs (DR1, DR2, and DR5 with the consensus sequence RGKTSA(N)1,2,5RGKTSA) in the promoters of all DEGs and found 36 genes (Table S10). The intersection of these two resulting gene lists revealed the well-known targets of RXRA, CYP26A1, which oxidizes retinoic acid cytochrome, and RARB, the retinoic acid receptor beta, which acts as a suppressor of cell growth. RARB activation correlates well with cell cycle arrest and the formation of a neuron-like phenotype. Thus, the molecular phenotypes strongly suggest successful RA-induced differentiation of SH-SY5Y cell lines.

**Figure 8:**
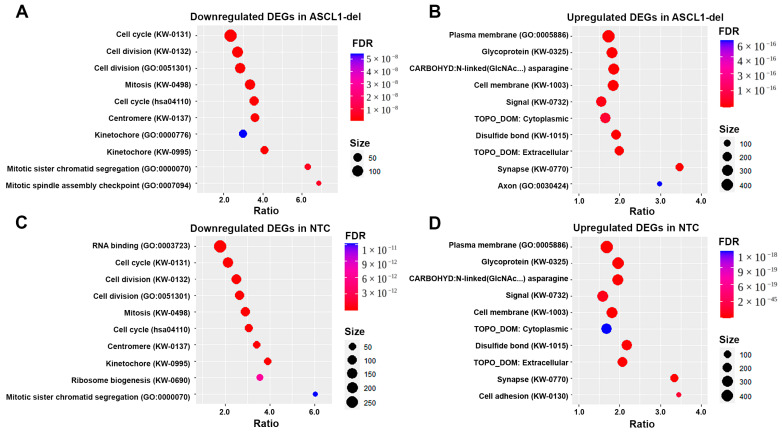
ASCL1 dysfunction has no significant effect on differentiated SH-SY5Y cells. (**A**) Enriched GO and UniProtKB categories for downregulated DEGs in ASCL1-del grown under normal conditions compared to RA-treated ASCL1-del. (**B**) Enriched GO and UniProtKB categories for upregulated DEGs in ASCL1-del grown under normal conditions compared to ASCL1-del treated with RA. (**C**) Enriched GO and UniProtKB for downregulated DEGs in NTC grown under normal conditions compared with RA-treated NTC. (**D**) Enriched GO and UniProtKB for upregulated DEGs in NTC grown under normal conditions compared with RA-treated NTC.

Subsequent analysis of the functional categories of DEGs showed that in both differentiated cell lines, the same categories are affected to essentially the same extent (Figure 8A–D). Thus, in both cell lines, the centromere, kinetochore, cell cycle, and cell division categories are equally downregulated (Figure 8A,C), indicating suppression of cellular processes associated with cell division (mature neurons are non-dividing cells). Both cell lines are characterized by the upregulation of the “synapse” category (Figure 8B,D), which is consistent with the observed morphological changes in the cultures (Figure S2). In addition, *ASCL1* mRNA levels were reduced in NTC (Table S4) but not altered in ASCL1-del (Table S5, *ASCL1* is absent in DEGs). Taken together, these data suggest that ASCL1 is normally repressed during neuronal differentiation, so its dysfunction does not have a significant negative effect on this process.

Functional knockout of *ASCL1* compared to NTC resulted in altered expression of 36 genes after RA-induced differentiation (*VIM*, *HEBP2*, *NNAT*, *GAL*, *ATP2B1*, *PLXNA2*, *NOS1*, *SCD*, *SEZ6L*, *NXT2*, *SPARC*, *IGFBP5*, *RGS4*, *TNFRSF10B*, *IFI6*, *ASCL1*, *NTRK2*, *HSD17B12*, *GUCY1A2*, *CNTNAP5*, *CLSTN2*, *DHRS3*, *STX3*, *COL3A1*, *LDLRAD4*, *CLCN5*, *INSM1*, *YES1*, *BASP1*, *SSTR2*, *ANXA2*, *INSIG1*, *S100A10*, *NRAP*, *LINC01013*, and *LOC107985953*) (Figure 3D). Eighteen of these DEGs are shared with NTC (Figure 3D) and have the same direction of change (upregulation), with the exception of *PLXNA2* (downregulation). Another group of ASCL1-specific DEGs includes a subset of genes that are silenced in ASCL1-del in standard culture and activated after RA treatment but that do not reach the level of the NTC cell line. These genes include *nNOS*, or *NOS1*, which encodes a neuronal nitric oxide synthase that produces NO, which in turn acts as a neurotransmitter and regulates synapse formation [36]; *NTRK2*, which encodes neurotrophic receptor tyrosine kinase 2, TrkB, which binds BDNF; *CLSTN2* (Calsyntenin 2), which encodes a postsynaptic adhesion molecule involved in synapse formation [37]; and *CNTNAP5*, which encodes contactin-associated protein family member 5 and is associated with dyslexia [38] and SZ [39]. All these genes are involved in memory and learning processes. Thus, our data suggest that ASCL1 functions in mature neurons are likely restricted to the regulation of genes involved in neuroplasticity.

## 3. Discussion

We found that functional knockout of ASCL1 in the model cell line SH-SY5Y leads to the deregulation of genes associated with SZ and other neurodevelopmental disorders. At the cellular level, genes affected by ASCL1 dysfunction constitute the signatures of GABAergic neuronal precursors and GABAergic neurons and affect synapse formation and function. At the molecular level, ASCL1-dependent deregulated genes are associated with cell division, cell signaling, and membrane-bound proteins, including intercellular contacts. Overall, our data suggest that ASCL1 dysfunction contributes to SZ development at cellular stages preceding neuronal differentiation mainly by slowing cell proliferation and impeding the formation of neuronal signatures and acts on mature neurons by negatively affecting neuroplasticity.

SH-SY5Y was derived from the SK-N-SH cell line, which was established from a biopsy of a patient with neuroblastoma [40]. In neuroblastoma, *ASCL1* appears to be under direct regulation by the LMO1 and MYCN oncogenes, and its overexpression correlates with poorer survival of patients with tumors [26]. Despite this, enrichment for disease-associated DEGs in the ASCL1-del mutant line showed that they are associated with colorectal cancer (Figure 5A,B). This is consistent with the literature evidence that the ASCL family of TF Is involved in the pathogenesis of cancer, including colorectal cancer [41]. Moreover, several genes from the G2M downregulated gene set (Figure 6A and Figure 7A), including *MKI67*, *MCM2*, *CCND1*, and their STRING-interacting genes *MECOM*, *TYMS*, and *MTHFR*, are associated with neoplasms [42,43]. These genes serve as markers of proliferation and appear to be controlled by ASCL1 in cancer cells. Nevertheless, the number of cancer-associated DEGs is much smaller than the number of genes associated with SZ and other neurodevelopmental disorders (BD and depression), which confirms the validity of this cellular model for further studies of SZ.

GSEA showed that functional knockout of *ASCL1* leads to the downregulation of a number of GABAergic midbrain signature genes (Figure 6). This is consistent with the fact that ASCL1, together with its target gene DLX1 encoding the Notch ligand, are important for the differentiation of GABAergic neurons. [44]. Given the important role of GABA-mediated neurotransmission [45,46,47,48,49] and the decreased activity of GABAergic neurons in the pathogenesis of SZ as well as in BD patients [50], these results further support the validity of our model.

Morphological observations (Figure S2) and gene enrichment analysis (Figure 8) suggest that differentiation of SH-SY5Y into a neuron-like phenotype is not impaired by the lack of *ASCL1*. This may be due to up-regulation of the TF NEUROG2, which has been shown previously to rescue the differentiation of ASCL1-deficient cells [15]. NEUROG2 has also been shown to form complexes with RA receptors in spinal cord motor neurons, where they activate gene expression by inducing H3/H4 acetylation via CBP histone acetyltransferase [51]. Our data also show that expression of the RA receptors RXRA and RARB, involved in the regulation of NEUROG2 expression [52], is not significantly affected by ASCL1 dysfunction, allowing the RA-induced differentiation of SH-SY5Y. However, with a lack of *ASCL1*, expression levels of *NOS1*, a known marker of GABAergic interneurons, as well as *NTRK2*, *CLSTN2*, and *CNTNAP5*, which are involved in neuroplasticity, are reduced. This suggests that ASCL1 dysfunction in GABAergic neurons may lead to memory impairment, which is a cognitive symptom observed in SZ patients [53].

We also found increased ASCL1 transcription in cells devoid of ASCL1 protein, suggesting direct or indirect regulation of ASCL1 via a negative feedback regulatory loop. This is consistent with the oscillatory nature of ASCL1 expression, the rate of which varies along a sinusoid with a period of 2–3 h, which provides a process of lateral inhibition in neurodevelopment [12]. We also observed that the *PAH* gene overlapping with *ASCL1* is significantly upregulated (Table S2). It is likely that the CRISPR/Cas9-deleted region contains a regulatory motif required for *PAH* repression. This observation is of particular interest given that the phenylalanine hydroxylase encoded by the *PAH* gene converts phenylalanine to tyrosine, a precursor of dopamine [54]. In turn, an imbalance in dopamine metabolism is considered to be one of the key factors involved in the pathophysiology of SZ [55]. Moreover, some *PAH* polymorphisms are associated with paranoid delusions and hallucinations [56].

Downregulated DEGs are enriched in REST binding sites according to ChIP-seq ENCODE results (Table 1, Figure 4A). REST (also called NRSF), a major silencing TF, is involved in the repression of a large number of neuron-specific genes involved in synaptogenesis, axonal pathway finding, synaptic plasticity, and structural remodeling [57]. In addition, unlike ASCL1, whose expression increases during neuronal differentiation, REST expression, which is high in embryonic stem cells, decreases with the onset of neuronal differentiation [58]. Similar to ASCL1, REST acts through chromatin remodeling [59]. Hence, we hypothesize that an indirect antagonism may exist between REST and ASCL1, affecting chromatin accessibility and, through this, controlling the activity of common target genes in neuronal cell precursors in the early stages of neurogenesis.

Given the important role of ASCL1 in the formation of specific neuronal patterns, it is possible that polymorphisms affecting ASCL1 activity could contribute to the cognitive and perceptual disturbances associated with SZ. To date, only two genetic variations potentially disrupting *ASCL1* have been found (rs533680685 (a 15 bp deletion in the coding region of the gene) and rs267606667 (a missense variant, c.52C > A, p.Pro18Thr)) in patients with congenital central hypoventilation syndrome [60]. It is unknown whether these patients have reached adolescence and, if so, whether they suffer from any psychiatric disorders. However, functional analysis has shown that mutant alleles of *ASCL1* impair noradrenergic neuron development in a cellular model [60]. In 2001, a case of a 5-year-old girl heterozygous for a large de novo deletion at 12q23 (involving the *IGF1*, *PAH*, and *ASCL1* genes) was described, resulting in phenylketonuria [61]. Although the patient has not been diagnosed with any psychiatric disorder, phenylketonuria is associated with brain developmental abnormalities leading to various neuropsychiatric manifestations, including SZ [62].

Understanding the ASCL1-mediated mechanisms associated with SZ suggests a possible improvement in SZ therapy. Our data suggest that functional knockout of *ASCL1* leads to the downregulation of midbrain GABA signature genes. Since GABAergic activity is reduced in the hippocampus of schizophrenic patients [50], our results suggest that increasing ASCL1 activity may be a potential therapeutic approach. ASCL1 levels are known to be negatively regulated by the Notch signaling pathway [63]. Consequently, Notch inhibitors can increase the level of active ASCL1 protein and stimulate neurogenesis or astrogenesis [64]. A number of such inhibitors have already successfully passed preclinical trials.

In conclusion, our results suggest that impaired ASCL1 function may lead to depletion of GABAergic neurons and/or reduced neuroplasticity. Enhancing ASCL1 activity with Notch pathway inhibitors may be a potential improvement to ER therapy.

## 4. Materials and Methods

### 4.1. Cell Culturing

The cell lines used in this study, SH-SY5Y and HEK293T, were kindly provided by E.B. Dashinimayev (Center for Precision Genome Editing and Genetic Technologies for Biomedicine, Pirogov Russian National Research Medical University). 

Cells were cultured in DMEM/F12 medium (Paneco LLC, Moscow, Russia) supplemented with 10% fetal bovine serum (Gibco, Waltham, MA, USA) with penicillin (50 U/mL) and streptomycin (50 μg/mL) (Paneco LLC). The medium was changed every 2–3 days. Once the cell layer reached a subconfluent state, cells were separated using a 0.05% trypsin-EDTA solution with Hanks’ salts (Paneco Ltd., Singapore) and passaged in a 1:4 ratio. Cultures were incubated in a humidified CO_2_ incubator MCO-18AC (Sanyo, Osaka, Japan) at 37 °C and 5% CO_2_.

### 4.2. Construction of Plasmids

A lentiviral vector expressing SpCas9 was constructed from two plasmids, pCW-Cas9 [65] and pEGFP-Puro [66]. The EGFP-Puro fusion marker was cloned into pCW-Cas9 using the restriction enzymes PspLI and BamHI. The resulting lentiviral vector pGPTet-Cas9 encodes SpCas9 under the control of tight-TRE (tetracycline response element) and the dual marker fused via T2A peptide in frame with advanced transactivator rtTA (eGFP-Puro-T2A-rtTA-Adv) under the control of the CMV promoter.

The two guide RNAs used for partial deletion of *ASCL1* were cloned in tandem as pLK05.sgRNA.EFS.tRFP [67]. To clone double sgRNAs, we created a Dual-sgRNA vector containing a PCR template for the sgRNA scaffold, a 55 bp long insert used to exclude recombination instability, and a U6 promoter. The expression cassette containing two spacers against *ASCL1* was amplified from the Dual-sgRNA vector using the primer pair ASCL1_S1_BsaI-F and ASCL1_S2_BsaI-F (Table S11). The resulting PCR product was digested with BsaI and cloned into pLK05.sgRNA.EFS.tRFP using BsmBI sites. The resulting pLK05-ASCL1 plasmid was used to generate the ASCL1-del mutant line. The coordinates of CRISPR targets in the *ASCL1* gene (GRCh38-hg38 genome version) were as follows: chr12:102,958,236–102,958,255 (Spacer 1) and chr12:102,958,333–102,958,352 (Spacer 2).

pLK05-NTC with a non-targeting sgRNA spacer (5′-GTTCTCTCTTGCTGCTGAAA-GCTCGA-3′) was kindly provided by E.B. Dashinimayev [68].

The pCheck reporter plasmid for ASCL1 translation assessment was designed based on the pEGFP-N1 vector (Clontech, Mountain View, CA, USA). 3xFLAG and T2A-(no-Met)GFP were amplified from plasmid pSpCas9(BB)-2A-GFP (PX458) [69] with primer pairs 3-FLAG_BsmBI_F and 3-FLAG_R and T2A_GFP_3FLAG_F and GFP_R, respectively, and then fused by PCR. The resulting PCR product was cut with BstAUI and BsmBI and cloned into pEGFP-N1 cut with HindIII and BstAUI. Because 3xFLAG-T2A-(no-Met)GFP does not have its own ATG codon, its translation depends only on the presence of ATG in the ORF, which can be cloned in-frame upstream of the reporter. The pEF1a(core)-tagRFP fragment from pLK05.sgRNA.EFS.tRFP fused to the bGH-polyA signal from PX458 was also cloned into the pCheck vector using pairs of the primers EF1a_PciI_F/RFP_rev_bGH_pA_R and bGH_polyA_F/bGH_PolyA_PciI_R. tagRFP is expressed independently of eGFP and is indicative of the presence of the pCheck vector within cells.

pCheck was used to create the pCheck-ASCL1-wt and pCheck-ASCL1-del plasmids, respectively, by the in-frame fusion of the *ASCL1* gene ORF with the 3xFLAG-T2A-(no-Met)eGFP reporter construct. *ASCL1* ORF was amplified from cDNA prepared from NTC or ASCL1-del control cells using the RevertAid RT Reverse Transcription Kit (Thermo Fisher Scientific, Waltham, MA, USA) according to the manufacturer’s instructions.

### 4.3. Transfections, Lentivirus Assembly, and Transductions

Transfections were carried out using Lipofectamine-3000 reagent (Invitrogen, Waltham, MA, USA) according to the manufacture’s protocol. HEK293T cells were grown until 50–70% confluency in ~1 day. In case of regular transfection, 2 µg of plasmid were used per 1 well of a 6-well plate in DMEM/F12 supplemented with 10% FBS. The efficacy of transfection was estimated based on the number of cells with detected fluorescence, using fluorescent microscopy in DPBS 24–48 h post-transfection.

In case of lentiviral assembly, the transfection was carried out with Lipofectamine-3000 in OptiMEM-I supplemented with 0.4 µg/mL Polybrene [70] (Sigma-Aldrich, St. Louis, MO, USA).

The target plasmid and pLP packaging plasmids were mixed and added in the following proportions: 5.8 µg target vector, 1.35 µg pLP1, 0.9 µg pLP2, and 1.33 µg pLP/VSVG. At 18–24 h post-transfection, the media was replaced with DMEM/F12 10% FBS, and the virus-containing supernatant was collected 48h and 72h post-transfection. The supernatant was diluted 2 times with fresh media, filtered through a 0.45-micron PES filter, and used immediately for the further transduction procedures.

Transduction of SH-SY5Y cells was carried out according to the Spinfection protocol [71] with the addition of 10 µg/mL polybrene. In case of transduction with pGPTet-Cas9, the cells were selected by puromycin treatment (2 mg/L, 2–3 weeks). In case of transduction with pLK05.sgRNA.EFS.tRFP-based gRNA vectors, no selection was applied due to the >90% efficiency of the procedure.

### 4.4. Differentiation of SH-SY5Y by RA Treatment

We used a modified protocol developed by Shipley et al., 2016 [72]. Cells were grown to 30% confluency on 10-cm plates in DMEM/F12 medium supplemented with 10% FBS. The medium was then changed to DMEM containing 1% FBS and 10 μM al-trans retinoic acid (ATRA), in which cells were incubated for 7 days, changing the medium every 48 h. The cells were then transferred to 10 cm matrigel-coated plates, and the medium was changed to Neurobasal medium containing B27, 10 μM ATRA, 50 ng/mL BDNF, and 20 mM KCl, in which the cells were maintained for 7 days. The differentiation procedure resulted in division arrest and changes in cell morphology, including the elongation of neurites and an increase in the number of synapses. 

### 4.5. Western Blotting

A 10 cm plate (~2.5 × 10^6^ cells) of each of the NTC and ASCL1-del lines was pelleted at 800× *g* at 4 °C, washed once with PBS, and lysed in RIPA buffer (50 mM Tris HCl (pH 7.4), 150 mM NaCl, 1% (*v*/*v*) NP-40, 0.5% (*w*/*v*) sodium deoxycholate, and 1 mM EDTA (pH 8.0), 0.1% SDS) for 30 min on ice, with DNA being further fragmented using a syringe. Samples were then loaded into a 15% SDS–polyacrylamide gel (~0.1 mg total protein per well) and run at 120 V for 3 h. Transfer to PVDF membrane was performed in Towbin buffer (25 mM Tris, 192 mM glycine, and 20% ethanol; pH 8.3) at 18 V for 90 min. The resulting membranes were blocked with 5% milk/TBST for 1 h and incubated with primary antibody (rabbit-anti-ASCL1, cat. # SAB2103889 (Sigma-Aldrich, USA), 1:1000 in 5% milk/TBST); rat-anti-α-tubulin, Cat. # ab6161 (Abcam, Cambridge, UK), 1:1000 in 5% milk/TBST)) at 4 °C for 16 h. Membranes were washed three times in TBST, then incubated with horseradish peroxidase-conjugated secondary antibodies (HRP-goat-anti-rabbit, Cat. # 111-035-144 (Jackson Immunoresearch Laboratories, West Grove, PA, USA), 1:5000 in 5% milk/TBST); goat-anti-rat, Cat. # 712-035-153 (Jackson Immunoresearch Laboratories), 1:1000 in TBST) at room temperature for 1 h, washed three times in TBST. Signals were detected using ECL Prime Western Blotting Detection Reagents (RPN2232, Cytiva, Marlborough, MA, USA) at 5 sec (for tubulin) and 15 min (for ASCL1). 

### 4.6. Fluorescence Microscopy

Fluorescent and phase contrast images were obtained with a Nikon Eclipse Ts2 microscope with a Nikon DS-Fi3 camera (Nikon, Tokyo, Japan), the cells being covered with DPBS buffer. GFP fluorescence was detected using fluorescence filter set #3 “GFP” (excitation 446–486 nm/emission 500–550 nm). The fluorescence of tagRFP was detected using fluorescence filter set #5 “mCherry” (excitation 542–582 nm/emission 604–678 nm).

### 4.7. RNA Isolation, RT-qPCR, and RNA-seq Library Preparation

RNA was isolated using the phenol-guanidine-isothiocyanate ExtractRNA reagent (Evrogen, Moscow, Russia) according to the manufacturer’s protocol. RNA was solubilized and stored in nuclease-free water treated with an RNase inhibitor (Syntol, Moscow, Russia). Residual genomic DNA was removed from samples using DNAase I (NEB, Ipswich, MA, USA). RNA integrity was checked using the Qubit RNA IQ Assay Kit (Thermo Fisher Scientific) on a Qubit 4 fluorimeter (Thermo Fisher Scientific). For further procedures, only samples with integrity and quality above 7.0 were used.

RT-qPCR was performed using the Luna Universal One-Step RT-qPCR Kit (NEB) on a Quant- Studio 7 Flex Real-Time PCR System (Thermo Fisher Scientific) according to the manufacturer’s protocol. The primers used for the qPCR are listed in Table S11.

RNA sequencing library preparation was performed in two steps. In the first step, polyadenylated RNA was enriched using the NEBNext Poly(A) mRNA Magnetic Isolation Module (NEB), and the resulting polyA RNA was used for subsequent library preparation using the NEBNext Ultra II Directional RNA Library Prep Kit for Illumina (NEB) according to the manufacturer’s protocols. Quality control (QC) and sequencing of the RNA libraries were outsourced to Macrogen Europe (The Netherlands). According to QC assessment, the average library size was 283 bp. NGS yielded ~12 M reads per sample (2 GB of data per sample).

### 4.8. RNAseq Data Analysis

Reads were trimmed using the FastP program [73]. Their quality was assessed using FastQC [74], and they were aligned to the Ensembl reference genome (GRCh38/hg38) [75] in pairwise mode using the STAR aligner program on the Yandex cloud server [76]. Alignments were subjected to deduplication using the Picard tool. Counts were performed using the HTseq program [77] at the gene level, and differential expression was assessed using DESeq2 in R (BioConductor package) [78]. GSEA analysis of DESeq2 results was performed using the fgsea BioConductor package (adjusted *p*-value < 0.05) [79] with searches against the MSigDB and Hallmark databases [28]. DisGeNET [23], Gene Ontology, and functional enrichment analyses were performed using the DAVID online enrichment service [24]. Each enrichment analysis was performed for the entire transcriptome obtained in the same experiment as the background. TF enrichment analysis was performed using the ChEA3 online enrichment service [80]. ChIP-seq data for ASCL1 were downloaded from http://chip-atlas.org/ [81] and overlapped at +/− 2 kb intervals from the transcription start site using the ‘bedtools intersect’ command [82].

### 4.9. Capture-C Data Analysis

Capture-C data for SH-SY5Y were obtained earlier in our laboratory [18]. Interactions were analyzed in the CHiCAGO library (BioConductor package in R) [83]. Visualization of Capture-C and GWAS data was performed in the IGV Genomic Browser [84].

## Figures and Tables

**Table 1:** Enrichment for TF binding sites among DEGs.

Set Name, TF (Cell Line)	*p*-Value	FDR	Odds Ratio
REST (HCT 116)	1.83 × 10^−9^	1.01 × 10^−6^	3.711
TCF7L2 (HEK293)	3.08 × 10^−5^	2.12 × 10^−3^	1.734
GATA3 (SK-N-SH)	8.41 × 10^−5^	4.64 × 10^−3^	1.910
GATA2 (HUVEC)	2.93 × 10^−4^	1.35 × 10^−2^	1.623
CBX2 (K562)	4.91 × 10^−4^	2.08 × 10^−2^	1.600

## Data Availability

The datasets generated during and/or analyzed during the current study are available from the corresponding author on reasonable request.

## Supplementary Materials

The following supporting information can be downloaded at: https://www.mdpi.com/article/10.3390/ijms242115746/s1.
