# Supplementary figures and images for: ASCL1 Is Involved in the Pathogenesis of Schizophrenia by Regulation of Genes Related to Cell Proliferation, Neuronal Signature Formation, and Neuroplasticity
Source: Int J Mol Sci. 2023 Oct 30;24(21):15746. doi: 10.3390/ijms242115746 (PMC10648210; doi:10.3390/ijms242115746)

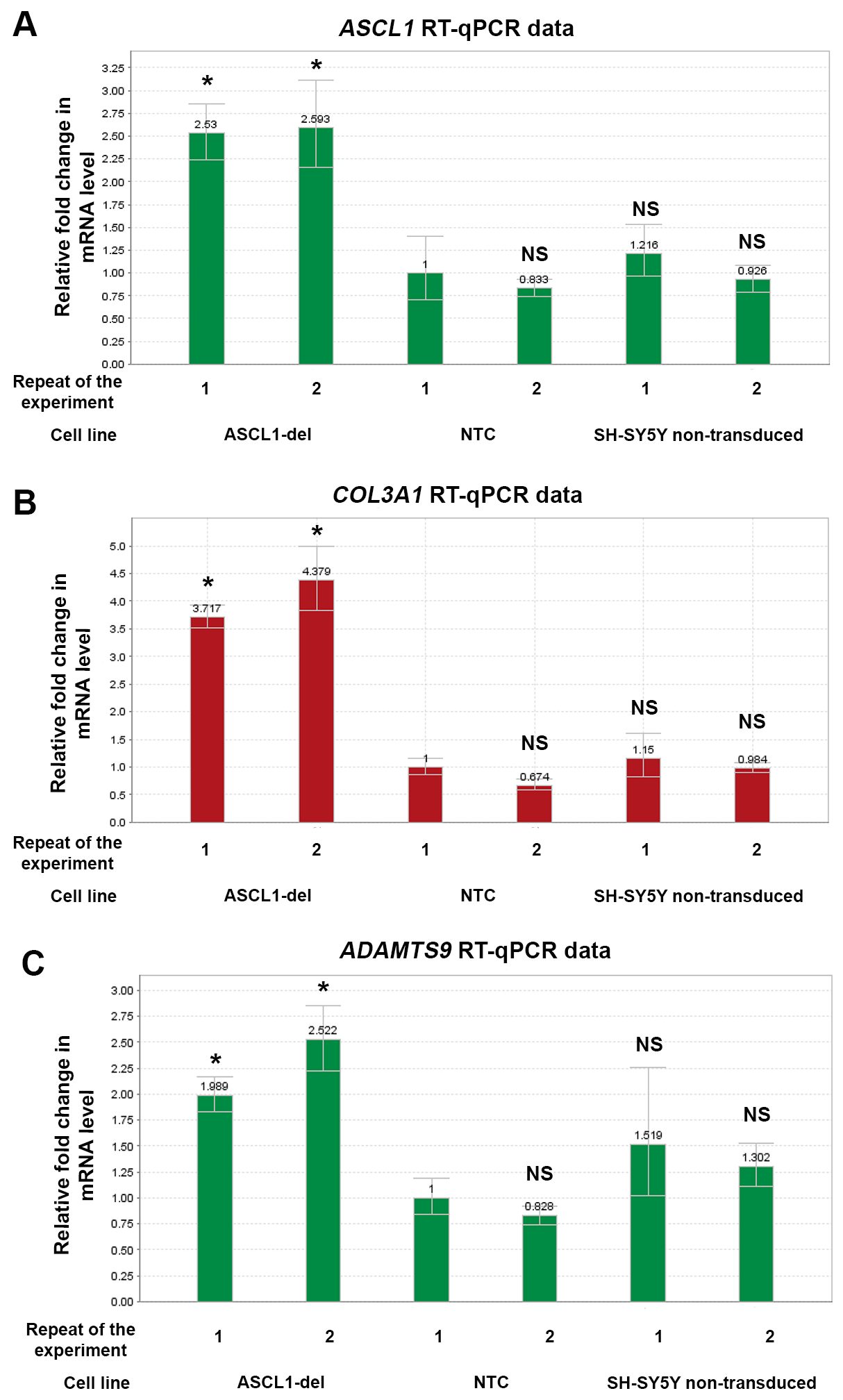

Supplement: Supplementary file 1 [file ijms-24-15746-s001.zip › Figure S1_.tif]

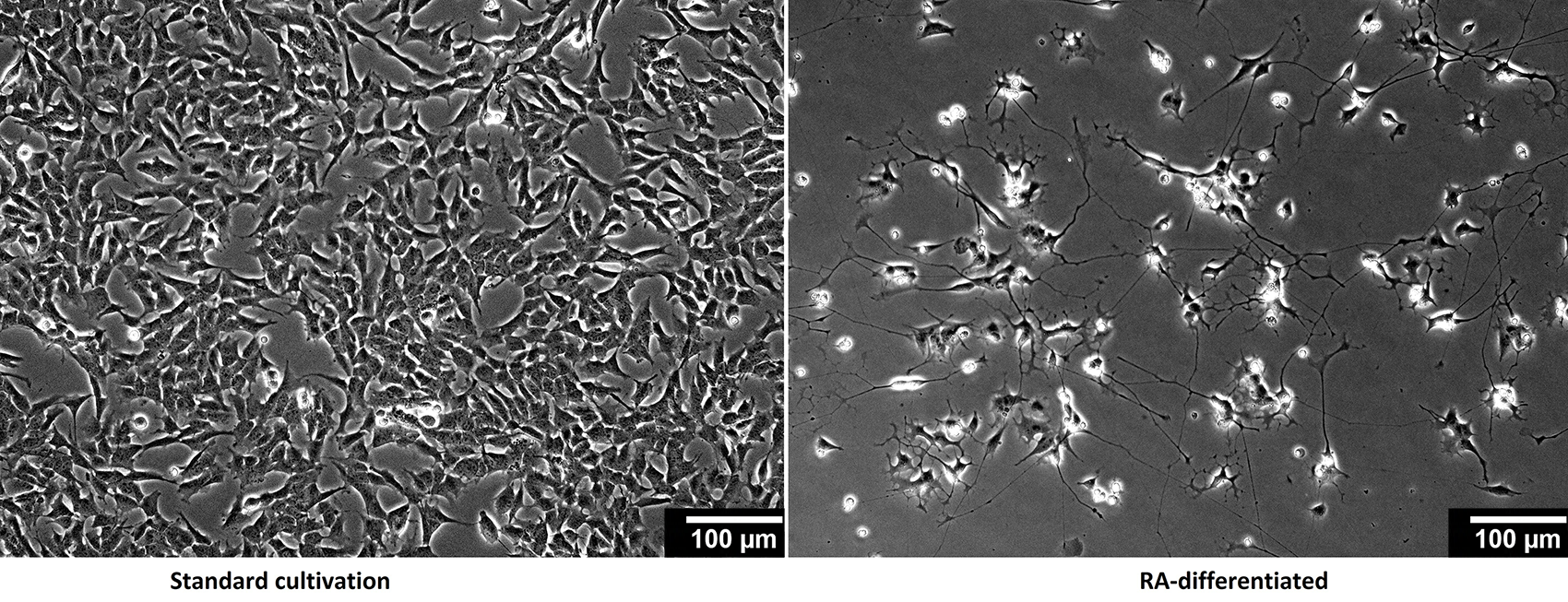

Supplement: Supplementary file 1 [file ijms-24-15746-s001.zip › Figure S2_.tif]
